# The relationship between the systemic inflammatory response, tumour proliferative activity, T-lymphocytic and macrophage infiltration, microvessel density and survival in patients with primary operable breast cancer

**DOI:** 10.1038/sj.bjc.6604667

**Published:** 2008-09-16

**Authors:** AM Al Murri, M Hilmy, J Bell, C Wilson, A-M McNicol, A Lannigan, J C Doughty, D C McMillan

**Affiliations:** 1 University Department of Surgery, University of Glasgow – Faculty of Medicine, Royal and Western Infirmaries, Glasgow G31 2ER, UK; 2 University Department of Pathology, University of Glasgow – Faculty of Medicine Royal Infirmary, Glasgow G31 2ER, UK; 3 Department of Surgery, Wishaw General Hospital, Lanarkshire, UK

**Keywords:** primary breast cancer, albumin, Ki-67, inflammatory cells, microvessel density, survival

## Abstract

The significance of the inter-relationship between tumour and host local/systemic inflammatory responses in primary operable invasive breast cancer is limited. The inter-relationship between the systemic inflammatory response (pre-operative white cell count, C-reactive protein and albumin concentrations), standard clinicopathological factors, tumour T-lymphocytic (CD4+ and CD8+) and macrophage (CD68+) infiltration, proliferative (Ki-67) index and microvessel density (CD34+) was examined using immunohistochemistry and slide-counting techniques, and their prognostic values were examined in 168 patients with potentially curative resection of early-stage invasive breast cancer. Increased tumour grade and proliferative activity were associated with greater tumour T-lymphocyte (*P*<0.05) and macrophage (*P*<0.05) infiltration and microvessel density (*P*<0.01). The median follow-up of survivors was 72 months. During this period, 31 patients died; 18 died of their cancer. On univariate analysis, increased lymph-node involvement (*P*<0.01), negative hormonal receptor (*P*<0.10), lower albumin concentrations (*P*<0.01), increased tumour proliferation (*P*<0.05), increased tumour microvessel density (*P*<0.05), the extent of locoregional control (*P*<0.0001) and limited systemic treatment (*P*⩽0.01) were associated with cancer-specific survival. On multivariate analysis of these significant covariates, albumin (HR 4.77, 95% CI 1.35–16.85, *P*=0.015), locoregional treatment (HR 3.64, 95% CI 1.04–12.72, *P*=0.043) and systemic treatment (HR 2.29, 95% CI 1.23–4.27, *P*=0.009) were significant independent predictors of cancer-specific survival. Among tumour-based inflammatory factors, only tumour microvessel density (*P*<0.05) was independently associated with poorer cancer-specific survival. The host inflammatory responses are closely associated with poor tumour differentiation, proliferation and malignant disease progression in breast cancer.

It is now recognised that the development of cancer and its progression is dependent on a complex interaction of the tumour and the host inflammatory response (Coussens and Werb, 2002; Vakkila and Lotze, 2004). Recently, the systemic inflammatory response, as evidenced by elevated circulating concentrations of C-reactive protein and hypoalbuminaemia, has been shown to be independently associated with poorer survival in patients with advanced disease (McMillan *et al*, 2001; Forrest *et al*, 2003) including breast cancer (Albuquerque *et al*, 1995; Zhang and Adachi, 1999; Al Murri *et al*, 2006). There is also some evidence that these acute-phase proteins have independent prognostic value in primary operable disease (McMillan *et al*, 2003, 2007) including breast cancer (Lis *et al*, 2003; Al Murri *et al*, 2007).

In animal models, at least, it would appear that the cell-mediated immune response is more important than humoural immunity in preventing the progression of cancer, and there is some evidence that cell-mediated immunity can bring about tumour regression. The principal cells involved in the cell-mediated response are T lymphocytes and macrophages (O'Sullivan and Lewis, 1994; Lee *et al*, 1996, 2006; Ogmundsdottir, 2001). However, this immune response, in particular the local environment of cytokines, proteases, angiogenic/growth factors and the resulting systemic inflammatory response, may, in turn, stimulate tumour growth and metastasis (O'Sullivan and Lewis, 1994; Leek *et al*, 1996; Yu and Rak, 2003; Lin and Pollard, 2007).

A number of studies have observed that, in breast tumours, there is a diffuse infiltrate of T lymphocytes and macrophages (Lee *et al*, 1996, 2006). It is also observed that there is an association with better outcome in patients with a moderate or marked diffuse inflammatory pattern in the subgroup of high-grade cases (Pupa *et al*, 1996; Lee *et al*, 2006). Recently, the use of immunohistochemical techniques to reliably identify and assess tumour-infiltrating T lymphocyte subsets and macrophages has led to renewed interest in the relationship between the tumour inflammatory infiltrate and cancer-specific survival in a variety of common solid tumours. With reference to tumour T-lymphocytic infiltration, a significant association with survival has been shown in renal (Bromwich *et al*, 2003), prostate (McArdle *et al*, 2004), colorectal (Canna *et al*, 2005; Galon *et al*, 2006) and head and neck cancers (Badoual *et al*, 2006). However, few studies have examined the association between tumour CD4+/CD8+ T-lymphocytic infiltration and/or CD68+ macrophage infiltration and survival in patients with primary operable breast cancer (Griffith *et al*, 1990; Wintzer *et al*, 1991; Leek *et al*, 1996; Toi *et al*, 1999; Tsutsui *et al*, 2005).

Griffith *et al* (1990) and Wintzer *et al* (1991) have both reported that disease-free survival and overall survival in breast cancer patients were not influenced by the tumour infiltration of any lymphocyte subset. However, these were relatively small studies of less than 80 patients. In contrast, different monocyte subsets appeared to be associated with either good or poor disease-free survival (Toi *et al*, 1999). Furthermore, in studies between 100 and 250 cases, there was conflicting evidence as to whether or not CD68+ macrophage infiltration was superior to microvessel density in predicting disease-free survival (Griffith *et al*, 1990; Wintzer *et al*, 1991; Toi *et al*, 1999; Tsutsui *et al*, 2005).

Therefore, the inter-relationship between local and systemic inflammatory responses and its prognostic significance in patients with primary operable breast cancer remains unclear. The aim of this study was to examine the relationship between circulating concentrations of C-reactive protein and albumin, tumour infiltration of T-lymphocyte sub-populations and macrophages and survival in patients who had undergone potentially curative surgical resection for invasive primary operable breast cancer.

## Patients and methods

Patients with histologically proven invasive primary operable breast cancer presenting consecutively to two hospitals (Western Infirmary, Glasgow and Wishaw General Hospital, Lanarkshire) in the west of Scotland between June 2001 and December 2002 and who had a pre-operative measurement of C-reactive protein and albumin (*n*=168) were studied prospectively.

Clinicopathological data included the age, deprivation category, histological type, tumour size, grade, lymph node status and oestrogen and progesterone receptor status. The type of surgery and the use of adjuvant treatment (chemotherapy, hormonal therapy and radiotherapy) were recorded.

The extent of deprivation was derived from the 1991 census, using the post-code of residence at diagnosis (Carstairs and Morris, 1991). The results are presented by amalgamating the seven categories into three groups: affluent (categories 1 and 2), intermediate (categories 3–5) and deprived (categories 6 and 7).

Routine pre-operative laboratory measurement of C-reactive protein, albumin and white cell count was carried out. At this time, no patient showed clinical evidence of infection or other inflammatory conditions. The coefficient of variation for these measurements was less than 10% as established by routine quality control procedures. The limit of detection of C-reactive protein concentration assay was 6 mg l^−1^, with the upper limit of normal values being ⩽10 mg l^−1^.

The study was approved by the local research ethics committees.

### Methods

Blocks from the primary tumour were fixed in 10% buffered formalin in saline and embedded in paraffin wax. One representative block of tumour was selected for each patient. Serial individual sections (4 *μ*m) were cut and mounted on slides coated with aminopropyltriethoxysilane for the immunohistochemistry of Ki-67 (proliferative index), CD34+ (microvessel density), CD68+ (tumour-associated macrophages) and CD4+ and CD8+ (T lymphocytes).

### Immunohistochemistry

Appropriate positive controls were included in each run. Negative controls were omission of the primary antibody.

#### Ki-67

Sections were immunostained using the peroxidase-based Envision technique (Dako, Cambridgeshire, UK) as described earlier (McNicol *et al*, 1997). The primary antibody for Ki-67 was mouse monoclonal antibody (Dako) at a dilution of 1 : 500.

#### CD34+

Sections were immunostained using the peroxidase-based Envision technique (Dako). The primary antibody for CD34+ was mouse monoclonal antibody (Novocastra, Newcastle upon Tyne, UK) at a dilution of 1 : 50.

#### CD68+

Sections were immunostained using the peroxidase-based Envision technique (Dako). The primary antibody for CD68+ was mouse monoclonal antibody (Dako) at a dilution of 1 : 200.

#### CD4+ and CD8+ T lymphocytes

Sections were immunostained using the peroxidase-based Envision technique (Dako) as described earlier (Bromwich *et al*, 2003). The primary antibody for CD4+ was mouse monoclonal antibody (Vector, Peterborough, UK) at a dilution of 1 : 10 and that for CD8+ was mouse monoclonal antibody (Dako) at a dilution of 1 : 1000.

### Morphometry

#### Ki-67

**Figure 1:**
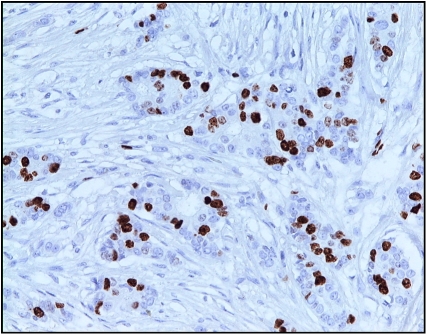
Ki67 immunohistochemical staining in invasive breast cancer ( × 200).

The percentages of Ki-67-reactive tumour cells were evaluated at a magnification of × 400 (Figure 1) by scoring a minimum of 1000 tumour cells in randomly selected fields (Ki-67 labelling index).

#### CD34+

**Figure 2:**
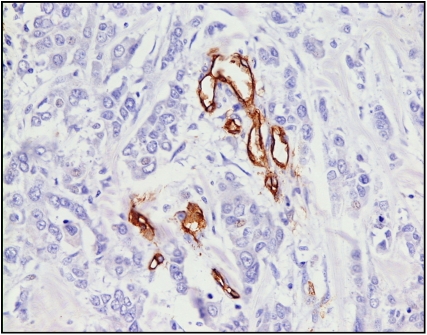
CD34+ immunohistochemical staining in invasive breast cancer ( × 200).

Quantitative analysis of the microvessel density was performed by selecting the three most vascular areas (hot spots), where the highest numbers of discrete microvessels were stained, at low powers ( × 40 and × 100; Figure 2). Counting of discrete vessels was performed with a magnification of × 200, using a 25-point Chalkley grid as described by Hansen *et al* (2000a, 2000b).

#### CD68+, CD4+ and CD8+

**Figure 3:**
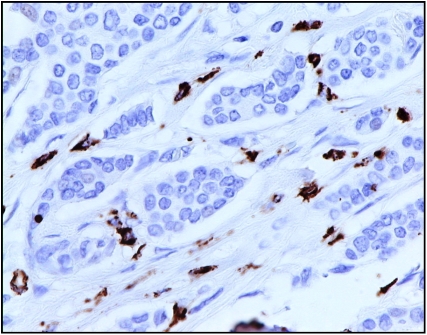
CD68+ immunohistochemical staining in invasive breast cancer ( × 400).

**Figure 4:**
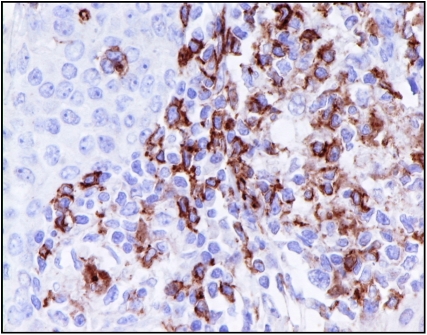
CD4+ immunohistochemical staining in invasive breast cancer ( × 400).

**Figure 5:**
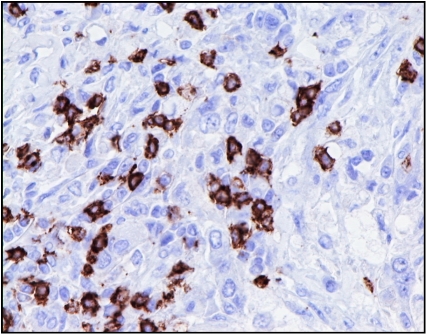
CD8+ immunohistochemical staining in invasive breast cancer ( × 400).

Quantitative analysis of the tumour-associated macrophages (CD68+; Figure 3) and lymphoid infiltrates (CD4+; Figure 4 and CD8+; Figure 5) was performed using a point counting method (Anderson and Dunnill, 1965) with a random sampling technique. With this method, the volume occupied by any given component (volume density) is expressed as a percentage of the total volume of the tissue. A 100-point ocular grid was used at a magnification of × 400 and 30 fields were counted per case for CD68+, CD4+ and CD8+ immunopositive cells.

Only fields containing tumour (including tumour nest and surrounding tissues stroma) were counted. Any normal tissue on the slide was excluded from the analysis. All the cases were counted by the author AMAM. For the purpose of assessing inter-observer reproducibility, a second observer (MH) and JB independently scored the slides for the tumour microvessel density (CD34+) and tumour-associated macrophages (CD68+), and T lymphocytes (CD4+ and CD8+) respectively. The observers were blinded to the clinical outcome of the patient.

### Statistics

Data are presented as median and range. Grouping of the laboratory variables was carried out using standard thresholds (Goldwasser and Feldman, 1997; McMillan *et al*, 2001). For the purpose of analysis, the tumour Ki67 proliferative index, tumour-associated macrophages (CD68+) and T lymphocyte subset populations (CD4+ and CD8+) were grouped by tertiles, and microvessel density (CD34+) was grouped by vascular grade based on Chalkley mean count with cutoff points at 5 and 7 as described by Hansen *et al* (2000a, 2000b). The relationships between these and other variables were analysed using the Mantel–Haenszel (*χ*^2^) test for trend and Spearman rank correlation as appropriate.

Survival analysis was performed using the Cox proportional hazard model. Multivariate survival analysis was performed using stepwise backward procedure to derive a final model of the variables that had a significant independent relationship with survival. To remove a variable from the model, the corresponding *P*-value had to be a greater than 0.10. Deaths up to the end of March 2008 were included in the analysis. Analysis was performed using SPSS software (SPSS Inc., Chicago, IL, USA).

## Results

The baseline clinicopathological characteristics of the patients with primary operable breast cancer (*n*=168) are shown in Table 1. One hundred and thirty-six (81%) patients were over 50 years of age, and 49 (29%) were in the most deprived categories 6 and 7.

Of the 168 patients, 142 (85%) patients had ductal carcinoma, 100 (60%) had a tumour less than 2 cm and 139 (83%) had a grade II/III tumour. Ninety-four (56%) patients had no axillary lymph node involvement. Thirty-five patients (21%) had oestrogen receptor-negative tumours.

Before surgery, the majority had white cell count, albumin and C-reactive protein concentrations in the normal range (96, 100 and 85% respectively). C-reactive protein concentration was correlated with albumin concentration (*r*_s_=−0.24, *P*=0.003) but not white cell count (*r*_s_=0.13, *P*=0.100).

In all, 162 (97%) patients received adjuvant treatment in the form of endocrine therapy and/or chemotherapy.

The inter-relationships between clinicopathological characteristics are shown in Table 2. In all patients, high tumour grade was positively associated with negative hormonal receptor status (*P*<0.001), high Ki-67 labelling index (*P*<0.001) and high expression of CD34+ (*P*<0.01), CD68+ (*P*<0.05), CD4+ (*P*<0.05) and CD8+ (*P*<0.05) T lymphocytes. Similarly, Ki-67 labelling index was positively associated with CD34+ (*P*<0.001), CD68+ (*P*⩽0.001), CD4+ (*P*<0.001) and CD8+ (*P*<0.01) T lymphocytes. Negative hormonal receptor tumours were positively associated with lower albumin concentration (*P*<0.05), high Ki-67 labelling index (*P*<0.001) and the presence of CD68+ (*P*<0.05) and CD8+ (*P*<0.05) T lymphocytes. An elevated C-reactive protein concentration was positively associated with the expression of CD34+ (*P=*0.05) and the presence of CD4+ T lymphocytes (*P*<0.05).

Microvessel density CD34+ was positively associated with the presence of CD68+ (*P*<0.01) and CD4+ T lymphocytes (*P*<0.05). Tumour-associated macrophages CD68+ were positively correlated with tumour CD4+ (*P*<0.01) and CD8+ (*P*<0.001) T lymphocytes. Tumour CD4+ T lymphocytes were also positively associated with CD8+ T lymphocytes (*P*<0.001).

The minimum follow-up was 64 months and the median follow-up of the survivors was 72 months. During this period, 18 died of their cancer and 13 of inter-current disease. On univariate survival analysis (Table 3), tumour size (*P*<0.10), lymph node involvement (*P*<0.0001), hormone receptor status (*P*<0.10), albumin (*P*<0.01), Ki-67 (*P*<0.05), microvessel density CD34+ (*P*<0.05), locoregional treatment (*P*<0.0001) and systemic treatment (*P*⩽0.01) were significantly associated with cancer-specific survival. On multivariate analysis of these significant covariates, albumin (HR 4.77, 95% CI 1.35–16.85, *P*=0.015), locoregional treatment (HR 3.64, 95% CI 1.04–12.72, *P*=0.043) and systemic treatment (HR 2.29, 95% CI 1.23–4.27, *P*=0.009) were significant independent predictors of cancer-specific survival. When albumin was excluded from the multivariate analysis, only locoregional treatment (HR 8.85, 95% CI 2.85–27.41, *P*<0.001) and systemic treatment (HR 2.09, 95% CI 1.15–3.81, *P*=0.016) were independently associated with poorer cancer-specific survival.

On univariate survival analysis (Table 3), age (*P*<0.10), tumour size (*P*<0.10), lymph node involvement (*P*<0.05), albumin (*P*<0.01), microvessel density CD34+ (*P*<0.10), locoregional treatment (*P*<0.01) and systemic treatment (*P*<0.10) were significantly associated with overall survival. On multivariate analysis of these significant covariates, age (HR 11.35, 95% CI 1.53–84.14, *P*=0.018) albumin (HR 3.58, 95% CI 1.56–8.20, *P*=0.003), locoregional treatment (HR 2.67, 95% CI 1.24–5.72, *P*=0.012) and systemic treatment (HR 1.60, 95% CI 1.06–2.41, *P*=0.025) were significant independent predictors of overall survival. When albumin was excluded from the multivariate analysis, only age (HR 5.27, 95% CI 1.24–22.35, *P*=0.024) and locoregional treatment (HR 3.44, 95% CI 1.66–7.12, *P*<0.001) were independently associated with poorer overall survival.

When the tissue-based inflammatory factors alone, including T lymphocytes, tumour-associated macrophages, microvessel density and Ki-67 proliferation index, were considered in the multivariate analysis, only increased tumour microvessel density CD34+ (HR 2.42, 95% CI 1.16–5.03, *P*=0.018) was independently associated with poorer cancer-specific survival.

## Discussion

In this study, increased tumour grade and Ki-67 labelling index were associated with increased infiltration by CD68+ tumour-associated macrophages, CD4+ and CD8+ T lymphocytes and increased tumour microvessel density in patients with primary operable breast cancer. Furthermore, increased Ki-67 labelling index and microvessel density were associated with poorer cancer-specific survival. These results may be consistent with the concept that there is an active immune response to poor tumour cell differentiation that acts to increase the proliferative activity, angiogenesis and dissemination of the tumour in these patients (Pupa *et al*, 1996; Tsutsui *et al*, 2005; Lee *et al*, 2006; Lin and Pollard, 2007). Alternatively, it may reflect a more passive consequence of increased cytokine excretion from high-grade proliferating tumours that attracts macrophages and T lymphocytes and increases microvessel density.

Earlier studies have shown that tumour CD4+ T-lymphocyte infiltration was associated with poor outcome, independent of grade or stage, in patients with a variety of cancer including renal and prostate cancer (Bromwich *et al*, 2003; McArdle *et al*, 2004). However, in this study, the extent of tumour lymphocyte and macrophage infiltration *per se* was not a significant prognostic marker in determining disease outcome, consistent with earlier studies (Griffith *et al*, 1990; Wintzer *et al*, 1991; Vgenopoulou *et al*, 2003).

Recently, Lee *et al* (2006) in 700 patients with stage 1 and 2 breast cancer and a median follow-up period of nearly 10 years reported that, on simple staining with haematoxylin and eosin, there was a significant relationship between the extent of both macrophage and lymphocytic infiltration and cancer-specific survival. Although, moderate or marked diffuse inflammation was present in only 10% of tumours, only moderate or dense tumour inflammatory infiltrates were associated with a better prognosis in the subset of patients with grade 3 carcinomas.

The apparent discrepancies in the results of this study and those of Lee *et al* (2006) and some other earlier studies may reflect methodological differences, including the subsets of immune cellular infiltrates examined and the way in which the inflammatory infiltrates were assessed. In this study, the subsets of the tumour cellular infiltrates were identified by immunohistochemistry and the density was assessed using a point counting technique. This approach provided a more objective assessment and circumvents the problem of variation in distribution within an individual tumour. In addition, some earlier studies have not included the type of surgery and/or the adjuvant treatment received in their survival analysis. However, the relatively limited number of events and the relatively short follow-up period in our study should also be taken into account.

In this study, also consistent with earlier works, increased tumour Ki-67 labelling index (Veronese *et al*, 1993; Scholzen and Gerdes, 2000; Trihia *et al*, 2003; Tsutsui *et al*, 2005) and microvessel density (Hansen *et al*, 2000a, 2000b; Uzzan *et al*, 2004; Tsutsui *et al*, 2005) were significantly associated with poorer cancer-specific survival. It was of interest that tumour-associated macrophages, in addition to T lymphocytes, were subordinate to increased tumour microvessel density and Ki-67 proliferation index, which were independently associated with poorer cancer-specific survival. This would suggest that the reported prognostic value of tumour-associated macrophages is probably due to their positive involvement in tumour angiogenesis (Leek *et al*, 1996; Tsutsui *et al*, 2005) and proliferation (Jonjic *et al*, 1998).

In agreement with our recent study (Al Murri *et al*, 2007), when potentially curative locoregional and systemic treatment based on hormonal receptor status were included in the multivariate survival analysis, none of the potentially prognostic clinicopathological and tumour-based inflammatory factors were independently significant. This probably reflects the close association between the risk assessment and the treatment received and their relative impact on relapse and survival. Adjuvant chemotherapy, in addition to its direct cytotoxic effect on cancer cells, might also attenuate surgery-stimulated tumour cell proliferation and angiogenic surge possibly occurring at distant dormant or indolent micrometastases (Retsky *et al*, 2004). Furthermore, adjuvant chemoradiotherapy may be effective by virtue of its cellular immune suppression and modification of specific host immune-related mechanisms (Reizenstein *et al*, 1985; Stewart and Tsai, 1993).

The basis of the observation that albumin had independent prognostic value is not clear but it may be that chronic illness, reflected by a lower albumin (Goldwasser and Feldman, 1997), also impacts on cancer survival. Alternatively, as a lower albumin concentration was directly associated with hormone receptor-negative tumours, an unfavourable prognostic sign, it may, in part, reflect the biological functions of circulating albumin that include binding and transporting of hormones and growth factors (Margarson and Soni, 1998), inhibiting growth in the breast tumour-cell cytosol (Soreide *et al*, 1991) and tumour proliferation by modulating the activities of autocrine growth regulatory factors (Laursen *et al*, 1990).

Other intracellular signalling systems may have important functions in regulating cancer-cell survival and progression pathways in patients with primary operable breast cancer. For example, there is increasing evidence that nuclear factor-*κ*B and its associated pathways may be important in tumour progression in patients with endocrine-resistant and hormone-negative tumours (Zhou *et al*, 2005; Ciucci *et al*, 2006; Haffner *et al*, 2006).

In summary, the results of this show for the first time the inter-relationships between the pre-operative systemic inflammatory response, tumour-based factors and outcome in patients with primary operable breast cancer. The host inflammatory responses appear to be closely related to poor tumour proliferation and differentiation and malignant disease progression in primary invasive early-staged disease. Only pre-operative albumin concentration, locoregional and systemic treatments were independent predictors of cancer-specific survival.

## Figures and Tables

**Table 1:** The clinicopathological characteristics of patients with invasive primary operable breast cancer

**Clinicopathological characteristics**	**Patients (*n*=168)**
Age (⩽50/>50 years)	32/136
Deprivation (1–2/3–5/6–7)	23/96/49
Type (ductal/lobular/special type)	142/20/6
Size (⩽20/21–50/>50 mm)	100/68/0
Grade (I/II/III)	28/87/52
Involved lymph node (0/1–3/>3)	94/52/21
Hormonal receptor status (ER+ PR+/ER+ PR− or unknown/ER− PR− or unknown)	54/79/35
White cell count (10^9^ per l)^a^	7.1 (3.4–13.5)
White cell count (<8.5/8.5–11/>11 × 10^9^ per l)	123/34/8
Albumin (g l^−1^)^a^	44 (37–50)
Albumin (>43/⩽43 g l^−1^)	82/68
C-reactive protein (mg l^−1^)^a^	⩽6 (⩽6–66)
C-reactive protein (⩽10/>10 mg l^−1^)	143/25
	
Ki-67 (tertiles 1, 2, 3)^b^	6.2/15.5/37.2
CD34+ (⩽5/5–7/⩾7)	39/74/55
% Tumour-associated macrophages CD68+ (tertiles 1, 2, 3)^b^	2.90/5.05/7.70
	
*% Tumour T lymphocytes*	
CD4+ (tertiles 1, 2, 3)^b^	0.03/0.30/1.32
CD8+ (tertiles 1, 2, 3)^b^	0.27/0.73/2.23
	
Loco-regional treatment (mastectomy alone or conservation surgery+radiotherapy/mastectomy+radiotherapy)	125/43
Systemic treatment (ER-based treatment) (hormonal/hormonal+chemotherapy/chemotherapy/none)	80/53/29/5

ER=oestrogen receptor; PR=progesterone receptor.

a Median (range).

b Median.

**Table 2:** Inter-relationships between the clinicopathological characteristics in patients with invasive primary operable breast cancer

	**Involved lymph node (*P*-value)**	**Hormonal receptor status (*P*-value)**	**Albumin (*P*-value)**	**C-reactive protein (*P*-value)**	**Ki-67 (*P*-value)**	**CD34+ (*P*-value)**	**CD68+ (*P*-value)**	**CD4+ (*P*-value)**	**CD8+ (*P*-value)**
Grade (I/II/III)	0.109	<0.001	0.216	0.653	<0.001	0.006	0.027	0.030	0.035
Involved lymph node (0/1–3/>3)		0.843	0.150	0.554	0.504	0.106	0.079	0.360	0.633
Hormonal receptor status (ER+ PR+/ER+ PR− or unknown/ER− PR− or unknown)			0.047	0.804	<0.001	0.402	0.030	0.128	0.017
Albumin (>43/⩽43 g l^−1^)				0.362	0.548	0.405	0.193	0.927	0.386
C-reactive protein (⩽10/>10 mg l^−1^)					1.000	0.054	0.252	0.028	0.120
Ki-67 (tertiles 1, 2, 3)						<0.001	0.001	<0.001	0.004
CD34+ (⩽5/5–7/⩾7)							0.002	0.048	0.256
									
*% Tumour-associated macrophages*									
CD68+ (tertiles 1, 2, 3)								0.002	<0.001
									
*% Tumour T lymphocytes*									
CD4+ (tertiles 1, 2, 3)									<0.001

ER=oestrogen receptor; PR=progesterone receptor.

**Table 3:** Univariate survival analysis of patients with invasive primary operable breast cancer

**Clinicopathological characteristics**	**Cancer-specific HR (95% CI)**	**Survival *P*-value**	**Overall HR (95% CI)**	**Survival *P*-value**
Age (⩽50/>50 years)	4.40 (0.59–33.05)	0.150	3.73 (0.89–15.62)	0.072
Deprivation (1–2/3–5/6–7)^a^	1.15 (0.84–1.57)	0.371	1.22 (0.96–1.55)	0.111
Type (ductal/lobular/special type)	0.31 (0.05–2.06)	0.227	0.52 (0.18–1.51)	0.227
Size (⩽20/21–50/>50 mm)	2.45 (0.95–6.32)	0.064	1.91 (0.94–3.88)	0.073
Grade (I/II/III)	1.83 (0.88–3.81)	0.104	1.35 (0.78–2.32)	0.284
Involved lymph node (0/1–3/>3)	3.13 (1.70–5.79)	<0.001	1.67 (1.06–2.63)	0.026
Hormonal receptor status (ER+ PR+/ER+ PR− or unknown/ER− PR− or unknown)	1.81 (0.94–3.48)	0.076	1.48 (0.91–2.43)	0.116
White cell count (10^9^ per l)	0.91 (0.71–1.17)	0.478	1.05 (0.88–1.26)	0.568
White cell count (<8.5/8.5–11/>11 × 10^9^ per l)	1.11 (0.50–2.49)	0.797	1.42 (0.81–2.49)	0.216
Albumin (g l^−1^)	0.71 (0.60–0.85)	<0.001	0.80 (0.70–0.91)	<0.001
Albumin (>43/⩽43 g l^−1^)	6.44 (1.85–22.41)	0.004	3.64 (1.61–8.22)	0.002
C-reactive protein (mg l^−1^)	0.99 (0.92–1.06)	0.7353	0.98 (0.92–1.04)	0.517
C-reactive protein (⩽10/>10 mg l^−1^)	0.69 (0.16–2.98)	0.6151	0.58 (0.18–1.92)	0.375
				
Ki-67 (tertiles 1, 2, 3)	1.97 (1.06–3.68)	0.033	1.34 (0.86–2.08)	0.193
CD34+ (⩽5/5–7/⩾7)	2.36 (1.15–4.85)	0.019	1.50 (0.91–2.45)	0.110
% Tumour-associated macrophages CD68+ (tertiles 1, 2, 3)	1.47 (0.80–2.69)	0.210	1.56 (0.99–2.47)	0.057
				
*% Tumour T lymphocytes*				
CD4+ (tertiles 1, 2, 3)	0.97 (0.55–1.70)	0.913	1.11 (0.72–1.71)	0.629
CD8+ (tertiles 1, 2, 3)	0.92 (0.52–1.63)	0.770	1.16 (0.75–1.79)	0.514
				
Loco-regional treatment (mastectomy alone or conservation surgery+radiotherapy/ mastectomy+radiotherapy)	8.70 (3.09–24.44)	<0.001	3.17 (1.56–6.41)	0.001
Systemic treatment (ER-based treatment) (hormonal/hormonal+chemotherapy/ chemotherapy/none)	2.14 (1.27–3.60)	0.004	1.44 (0.95–2.16)	0.085

CI=confidence interval; ER=oestrogen receptor; HR=hazard ratio; PR=progesterone receptor.

a Individual deprivation categories were used in the statistical analysis. HR >1 trend towards worse survival with each incremental change, HR <1 trend towards better survival with each incremental change.
